# Promoting good health research practice in low- and middle-income countries

**DOI:** 10.3402/gha.v9.32474

**Published:** 2016-08-05

**Authors:** Yodi Mahendradhata, Jamila Nabieva, Riris Andono Ahmad, Patricia Henley, Pascal Launois, Corinne Merle, Christine Maure, Olaf Horstick, Varalakshmi Elango

**Affiliations:** 1 Center for Tropical Medicine, Faculty of Medicine, Gadjah Mada University, Yogyakarta, Indonesia; 2 Freelance Consultant, Heidelberg, Germany; 3 London School of Hygiene and Tropical Medicine, University of London, London, United Kingdom; 4 UNICEF/UNDP/World Bank/WHO Special Program for Research and Training in Tropical Diseases, Geneva, Switzerland; 5 World Health Organization, Geneva, Switzerland; 6 Institute of Public Health, University of Heidelberg, Heidelberg, Germany; 7 Freelance Consultant, Chennai, India

**Keywords:** capacity building, training, ethics, quality, developing countries

## Abstract

**Background:**

Good clinical practice (GCP) guidelines have been the source of improvement in the quality of clinical trials; however, there are limitations to the application of GCP in the conduct of health research beyond industry-sponsored clinical trials. The UNICEF/UNDP/World Bank/WHO Special Program for Research and Training in Tropical Disease is promoting good practice in all health research involving human through the Good Health Research Practice (GHRP) training program initiative.

**Objective:**

To report the results of piloting the GHRP training program and formulate further steps to harness GHRP for promoting good practices in all health research involving human, particularly in low- and middle-income countries (LMICs).

**Design:**

The objective of this training is to impart knowledge and skills for the application of ethical and quality principles to the design, conduct, recording, and reporting of health research involving human participants based on the level of risk, to ensure a fit-for-purpose quality system. This has been formulated into five sequential modules to be delivered in a 4-day course. Four courses have been organized in the pilot phase (2014–2015). The courses have been evaluated and assessed based on course feedback (quantitative and qualitative data) collected during course implementation and qualitative email-based pre- and post-course evaluation.

**Results:**

Participants were highly satisfied with the course content and its organization. The relevance and applicability of the course content resulted in positive feedback and an articulated willingness to adapt and disseminate the course. Action points to strengthen the training program have been identified, and showed the imminent need to develop a consensus with a broader range of key stakeholders on the final set of GHRP standards and means for implementation.

**Conclusions:**

There is an urgent need to harness the momentum to promote high-quality and ethical health research in LMICs through scaling up GHRP training and further development of GHRP principles into international standards.

## Background

Health research often involves human participants; hence, it is necessary to respect the rights, safety, and well-being of research participants and ensure that research is conducted with the best possible scientific rigor for generating reliable evidence to inform health policies. Several guidelines have been developed to promote good research practices, including the Declaration of Helsinki, the guidelines of the Council for International Organizations of Medical Sciences (CIOMS) and the World Health Organization (WHO), and the International Conference on Harmonization (ICH) Good Clinical Practice (GCP) guidelines. ICH–GCP is an international ethical and scientific quality standard for designing, conducting, recording, and reporting trials that involve the participation of human subjects (1). Compliance is intended to assure that the rights, safety, and well-being of participants are protected, and that trial data are credible (2). Hence, ICH–GCP has been the source of improvement in the quality of clinical trials; however, evidence has shown that there are several limitations to the application of and compliance to GCP in the conduct of academia and non-industry sponsored clinical trials (2–6). Limitations to the applicability of the GCP have been attributed to difficulties in the interpretation of the guidelines, the increased cost of conducting research, the overwhelming documentation process, and a focus on procedural aspects rather than science. In Europe, since the launch of the European Union Clinical Trials Directive, scientists have warned that the new requirements, and added paperwork and costs would hinder trials by academic scientists (7, 8).

Moreover, most health research falls outside the realm of the ICH–GCP regulatory requirement. Despite this fact, some funding agencies, publishers, and ethics committees increasingly expect GCP compliance for non-clinical trials’ research (2–4), likely due to the absence of an alternative guideline for conducting these other types of health-related research. In 2002, the WHO developed the *Handbook for Good Clinical Research Practice (GCP): Guidance for Implementation* (9) as an adjunct to WHO's guidelines for GCP for trials on pharmaceutical products (10). The handbook incorporated ICH–GCP and is intended to assist national regulatory authorities, sponsors, investigators, and ethics committees in implementing GCP for clinical research.

Unfortunately, the abovementioned guidelines were also found to be difficult to implement and contributed to only minor changes in low- and middle-income countries (LMICs) (3). Key limitations to the application of GCP to non-clinical trial health research are detailed in Box 1. It should be noted that even though GCP may not apply to all types of research involving human participants, the basic principles of ethics and quality are universally accepted as a means of ensuring the protection of human participants and the validity of research data and should be promoted. Therefore, there is a clear need to develop pragmatic and sensible guidance following the GCP principles to benefit the global health research community; a tool to assess the level of risk to research participants and ensure a fit-for-purpose quality system for individual research projects.

Box 1 **Key limitations to the application of GCP to non-regulatory human health research**

- Observance with rigid and blanket overarching guidelines aimed at randomized controlled trials of investigational medicinal products is required.
- Difficulties in interpretation exist.
- Compliance with GCP has led to spiraling costs of clinical research in countries that already have financial and human resource constraints; diverts scarce research funds towards compliance besides discouraging research in under resourced and under staffed health structures, where the need is the biggest.
- A rigid and onerous bureaucracy in the documentation and filing of more than 50 different documents, described as essential in the GCP guidelines, diverts the focus of the investigator from science and participant care to paperwork and administration.
- The application of processes like ‘monitoring’ and ‘auditing’, for example, to operational and implementation research puts tremendous pressure on the investigators to meet the GCP standards when not warranted, even though the added value has not been demonstrated.
- The norms prescribed for the design and contents of the protocol are not well suited to those health researches, which try to answer research questions using qualitative research methods.

Recognizing the need to enhance the knowledge and understanding of the research community regarding the basic concepts and principles of ethics and quality in all health research involving human participants, the UNICEF/UNDP/World bank/WHO Special Program for Research and Training in Tropical Disease (TDR) is promoting good practice in all types of health research involving human participants through the Good Health Research Practice (GHRP) training program initiative. This is the first step towards the development of guidelines to assure quality in health research involving human participation, particularly in LMICs. This training program has been developed in collaboration with the Regional Training Centers (RTCs) for Health Research supported by TDR. These RTCs are based in LMICs and have been competitively selected to develop a cadre of highly skilled health professionals through courses on the organization, management, and conduct of health research with a special emphasis on GHRPs and implementation research (11). We report here the results of piloting the GHRP training program and formulate further steps to harness GHRP for promoting good practices in all health research involving human, particularly in LMICs.

## Method

TDR brought together scientists with extensive experience in LMICs representing diverse areas of expertise in public health research, quantitative and qualitative research, ethics, quality management, and education to develop a short training course curriculum (Box 2). Formulated as a 4-day course, the primary objective of this training is to impart knowledge and skills for the application of ethical and quality principles to the design, conduct, recording, and reporting of health research involving human participants based on the level of risk, to ensure a ‘fit-for-purpose’ quality system. These principles have been formulated as GHRP principles (Box 3). The training adopted a methodology based on the experiential learning cycle (12) and following a step-by-step learning approach, similar to the WHO TDR entitled ‘Effective project planning and evaluation in biomedical research’ (13), a short training course that has been disseminated by RTCs in Africa, Asia, Latin America, and the Caribbean. During the course, participants apply the ethics and quality concepts and principles to concrete examples, allowing them to learn by ‘doing’ and ‘reflecting’. Short theoretical sessions are followed by extensive practical sessions, in which the participants work in small groups on their own research projects. In subsequent plenary sessions, each group shares its work and feedback for the benefit of all participants.

Box 2 **GHRP training framework**

- Module 1: Principles of research ethics and quality
- Module 2: Designing and planning the research
  - Study planning and management
  - Developing the protocol
  - Informed consent
  - Tools for collection and reporting of study data
  - Tools to facilitate study conduct and quality assurance and other essential documents
  - Study sites and study team
  - Research oversight
- Module 3: Conducting, recording, and monitoring the research
  - Informed consent procedure
  - Managing and analyzing the data
  - Quality system in research
- Module 4: Evaluating the research
- Module 5. Reporting and dissemination of the results

Box 3 **Key principles of Good Health Research Practices (GHRP)**

- **Ethics and quality** underpin all types of research involving human participants.
- **Risk assessment** should be done prior to and during the course of research with appropriate mitigation measures put in place.
- **Informed consent** should be appropriate for the study and in accordance with the cultural context of the study site.
- **Procedures** should be written in line with the study protocol to ensure the consistency and conformance of activities.
- **Staff qualified** through appropriate training, education, and experience will undertake roles in line with their qualification.
- Study activities should be well **planned and monitored** to assure the process and data quality.
- The **privacy** of the research participants and the confidentiality of all data acquired during the study should be duly protected.
- Research **results** and **reports** should be made publicly available.

Courses were organized in the pilot phase (2014–2015) (Table 1), which can be grouped according to their specific objectives and expected results. A pilot course in Heidelberg, Germany (April 2014), was implemented as a proof of concept to 1) test course material and the selected teaching approach; 2) revise the teaching material based on the participants feedback and facilitators experience; and 3) verify a potential demand in a course on ethics and quality standards of human health research. The course was attended by 28 post-graduate students working on their individual master's and doctoral studies at the Institute of Public Health, University of Heidelberg. Participants were broken into several working groups based on the research methods of interest, and each group had to select one study protocol from a list of individual proposals submitted by participants. The course in Heidelberg was conducted by four facilitators, who had participated in the development of the course materials and methods.

**Table 1:** Characteristics of participants in four courses

Course	Year	Participants	Participants’ origin	Academic background	Institutional background
Heidelberg	2014	28	Afghanistan, Bangladesh, China, Ethiopia, Germany, Guatemala, India, Indonesia, Mozambique, Myanmar, Nigeria, Rwanda, Taiwan, Tajikistan, Tanzania, USA, Zambia	17 master student, 11 PhD and post-doctoral students	N/A
Jogjakarta	2014	15	Indonesia, Philippines, Kazakhstan, Colombia	Master's and PhD	Faculty members and researchers
Jogjakarta	2015	15	Bangladesh, India, Indonesia, Nepal	Master's and PhD	Faculty members and researchers
Almaty	2015	13	Azerbaijan, Kazakhstan, Kyrgyzstan, Tajikistan, Uzbekistan	Master's and PhD	Faculty members, researchers, and ethics committees
Total in four courses		71			

Two courses were organized at the RTC at the Gadjah Mada University (GMU) in Jogjakarta, Indonesia (August 2014, July 2015), and aimed to 1) evaluate the course improvement in the pilot phase and its applicability; and 2) identify other training needs that can be addressed by expanding the GHRP course material or developing new courses. These courses were moderated by five facilitators; four were members of the course development team and one was a GCP trainer who was not previously involved in the course development. A course in Almaty, Kazakhstan, was organized by the RTC at the Astana Medical University and supported by TDR (May 2015) as a parallel dissemination process to 1) elicit a potential demand for the course in a region where awareness of international research principles and practice is quite limited (ex-Soviet Union countries); and 2) test the course in another language (Russian) – based on the assumption that most researchers in post-Soviet countries have limited access to and, hence, limited benefit from global scientific evidence that is mainly disseminated in English. This course was facilitated by six facilitators: three were members of the course development team, and three were Russian-speaking researchers who had taken part as participants in the previous courses.

Experience from the first course in Heidelberg suggested that the selection of participants based on groups of colleagues working on a single research project may ensure that the course is more effective and the teaching material more relevant. Hence, participants of the next three courses (Jogjakarta and Almaty) were selected based on research protocols submitted prior to the course; selected teams worked on their actual group proposals throughout the course.

In order to assess the course material, teaching methods, and participant satisfaction, an internal evaluation exercise was commissioned. The major evaluation aims intended to 1) synthesize course feedback provided by the participants of the four courses; and 2) based on the findings, develop recommendations for standardizing the GHRP materials and dissemination approach. The data set used for the evaluation comprises 1) course feedback (quantitative and qualitative data) collected during course implementation and 2) qualitative email-based pre- and post-course evaluation. The course feedback included 1) participants’ daily feedback and final evaluation of the course in Heidelberg (2014) and Jogjakarta (2014, 2015); 2) pre- and post-course assessment based on quantitative grading system, Jogjakarta (2014, 2015); 3) semi-structured interviews with Almaty course (2015) participants; and 4) facilitators’ notes made during and after courses in Heidelberg (2014) and Almaty (2015).

Qualitative pre- and post-course evaluation focused on four major categories around the course: knowledge, skills, practical applicability, and further capacity-building needs. In the pre-course evaluation email, 1 week prior to courses in Almaty and Jogjakarta, participants were asked what kind of 1) knowledge and 2) skills they expected to obtain in the course; 3) where and how they would like to apply them; and 4) their other capacity development needs. In the post-course evaluation, 3 months later, participants were asked to share situations in which they had applied 1) knowledge; 2) skills obtained in the course; 3) what they had done differently as a result of new skills; and 4) their current training needs.

A two-fold analysis was applied to synthesize and interpret different sets of data. Data collected from the course feedback were reduced to three major categories: 1) course content and materials; 2) teaching methods and learning experience; and 3) course organization and duration. For each category, the data were further broken into the following subcategories: 1) what participants liked about the course; 2) what participants disliked or thought should be improved; and 3) participants’ practical suggestions and considerations. Analysis of the email-based pre- and post-evaluation data was based on predefined four categories.

Results from the quantitative pre- and post-course assessments in Jogjakarta (2014, 2015) were summarized in quartiles. The quartiles divided the data set into four equal groups, each group comprising a quarter of the data. The first quartile (Q1) is defined as the middle number between the smallest number and the median of the data set. The second quartile (Q2) is the median of the data. The third quartile (Q3) is the middle value between the median and the highest value of the data set.

Given the difference in objectives and approach applied, the evaluation findings are presented separately for each group of courses in the following section.

## Results

### First piloting in collaboration with the University of Heidelberg in Heidelberg, Germany

The overall feedback from the 28 participants on the course was positive, participants found the content very relevant and timely, and suggested that the GHRP or its elements should be included in the master's program as being practical for thesis research preparation and conduct.

The presented ethical principles and quality standards of health research involving human participants were reported to provide a valuable insight to areas that were generally seen as quite abstract. The training material was valued for a clear and logical structure; exercises to implement the lectures were seen as extremely practical. One of the participants for instance noted: ‘Very useful for my thesis research preparation …. The steps to plan and manage my research proposal were great, because it helps me to re-evaluate a lot of my proposals’**.

Participants appreciated the interactive, engaging, and friendly learning environment, as well as the facilitators’ capacities and encouraging support during the course. The opportunity to learn from a group of international ‘experts with real-life experience’ was seen as a unique opportunity. One participant explained: ‘The course was very intensive, but lively and interactive, a very friendly, cheerful environment, did not affect the flow and effectiveness of the learning process ….’

Reporting on aspects that could be improved, participants generally mentioned the intensity of the course and felt they needed more time to fully absorb the material and consolidate the new knowledge. Some participants believed that theory was overemphasized, and more case-studies and practical examples would foster a better understanding. Others felt the course was predominantly focused on clinical trials, hence, suggested the incorporation of more elements of public health research, specifically qualitative methods. One of the participants, for example, highlighted: ‘Trainers spoke too much about clinical trials; so, could not establish proper connection and was less relevant as we are not much into trials’**. Participants reported that the major difficulty was working in groups that mixed people with different academic background and areas of expertise.

To address participants’ feedback, the training material was revised to include more practical exercises after each learning session; make a fair distribution of input on non-clinical research aspects, with more focus on public health research; add a session on qualitative data collection and analysis methods. Finally, the participant selection approach is now based on the pre-submission of research protocols by groups of colleagues.

### Piloting in collaboration with GMU in Jogjakarta, Indonesia

The two courses conducted by the RTC at the GMU in 2014 and 2015 were attended by a total of 30 public health researchers. In each workshop, five teams of researchers from four countries worked on their group proposals, which made the learning process highly relevant and applicable. Quantitative evaluations by the participants are presented in Table 2. In general, the course was seen as a valuable systematization of crucial evidence and best practices in public health research.

**Table 2:** Participant's evaluation of the first and second good health research practice courses in Jogjakarta (scale: 1–5)

	Jogjakarta Course 1 (*n*=15)			Jogjakarta Course 2 (*n*=15)		
		
Category	Q1^a^	Q2 (Median)^b^	Q3^c^	Q1^a^	Q2 (Median)^b^	Q3^c^
Evaluation of learning experience						
Clear information about the training goal	4	4	5	5	5	5
Objectives of the module relates to present and future work	4	5	5	4	5	5
Appropriateness of contents in the module	4	4	5	5	5	5
Time allocation for each module is appropriate	4	4	4	4	4	5
Appropriateness of teaching methods	4	4	4.5	4	5	5
Appropriateness for application in future work	4.5	5	5	4	5	5
Demonstration materials and handouts	4	4	5	4	4	5
Evaluation of instructors/facilitators						
Readiness for teaching	4	4	5	4	5	5
Ability to transfer knowledge	4	4	5	4	5	5
Opportunity for students to ask questions and discuss in the class room and outside	5	5	5	4	5	5
Ability to motivate effective group work	4	4	5	4	5	5
Training organization and facilities						
Training hall	4	5	5	4	5	5
Refreshments	4	5	5	5	5	5
Organization	4	5	5	5	5	5

a First quartile

b Second quartile

c Third quartile.

Over 90% of participants found the training material very useful, specifically with regard to ethics and quality. Teaching was valued as a well-structured and step-by-step presentation of material with clear objectives for each session and practical exercises that ensured the proper absorption of material. Working on their own protocols throughout the course, including allocations of time for necessary revisions and improvements, was considered an extremely practical exercise. One participant, for instance, noted: ‘There is new material that I got from this course which is never given in other courses like GCP, GCLP, etc.; this overall material is relevant to our study project’.

Participants’ suggestions for improvement collected after the course in 2014 (including team-building exercises, more visual and graphic elements in slides, more planned time for group work, more focus on public health research rather than clinical trials to public health research) were incorporated in the training material used for the second course in 2015. The feedback from the second course suggests that the revised training material ensured a good balance between the technical input (lectures) and practical exercises (group work), as well as a fair combination of clinical and public health research principles. One of the participants highlighted, ‘Clear learning objectives for each sessions. The group work to implement what we learned. The open-ended questions during sessions made it interactive rather than a classroom type of setting’.

A follow-up evaluation conducted 3 months after the second course aimed to identify the applicability of the course content. The findings demonstrated that, in addition to general improved performance of routine responsibilities, participants applied the knowledge and skills obtained in the course for a wide range of purposes: counselling master's and doctoral students in the proper design of research protocols (40%); integrating GHRP elements into existing teaching curricula (25%); revising and improving the documentation of ethics committees (33%); and peer-reviewing manuscripts for journals (7%). Participants also reported having identified gaps and weaknesses in their teaching materials, methodologies, or the normative documents of their own institutions. One participant, for example, reported: ‘I now evaluate the quality of the research step by step, especially when developing a research proposal. I also tried to evaluate the research I have done in the past and to identify the things that need improvement in the future’.

Other capacity development and strengthening needs were another focus of the evaluation exercise. Training areas or topics reported as actual training needs can be grouped as 1) various sub-areas or extended elements of GHRP (such as research project management, qualitative research methods, the building and management of databases, and the identification of cultural determinants of ethics and public health research that can be developed as refresher trainings); and 2) areas beyond the scope of GHRP (such as advanced statistical analysis, report writing, proposal writing, team management, data management, global health, and training for ethics committees).

### Piloting in collaboration with Astana Medical University in Almaty, Kazakhstan

The workshop in Almaty was organized by the TDR-supported RTC at the Astana Medical University as a test of the GHRP course in the Russian language. Participants were provided with translated course material, theoretical sessions were supported by simultaneous translation, and the group work was facilitated in both the Russian and English languages. Thirteen public health researchers from four Central Asian countries (Kazakhstan, Kyrgyzstan, Tajikistan, and Uzbekistan) and Azerbaijan worked in four respective groups. The evaluation of course effectiveness and applicability was based on email-based pre- and post-course (3 months later) and an email survey; results showed a high rate of self-reported training needs: −85% of participants expressed a willingness to develop and continuously strengthen capacities in all aspects of public health research (85% of participants). Some participants suggested developing a course to train regional experts and trainers in GHRP, which would eventually institutionalize the course in a comprehensive and sustainable way. Most participants (77%) mentioned a significant gap in the knowledge and skills needed to conduct health research in compliance with international standards, hence, an interest in integrating the GHRP course practice or its elements in the existing curricula for post-graduate students. One participant reported: ‘I'm going to share my new knowledge and skills as a short training course for three target groups: 1) PhD and master's course students, 2) scientific mentoring professors, 3) members of the NEC [National Ethical Committee] and Bioethics Committee’.

Facilitators’ notes from these pilot courses suggest that there was progress in putting more emphasis on public health research, qualitative, and mixed method approaches. However, more efforts would still be needed to ensure that these are actually addressed adequately in each session, with practical examples. Facilitators also noted that managing time for particular sessions was difficult, highlighting the need to review the essential contents of each session, as well as anticipate issues that stimulate lengthy discussions. The use of simultaneous translation in the course in Kazakhstan was found to be particularly challenging by facilitators.

## Discussion

The course evaluation suggests a potentially great demand for GHRP, alongside high participant satisfaction with the course content and its organization. The relevance and wide applicability of the GHRP course content resulted in exclusively positive feedback and an articulated willingness to adapt and disseminate the course in Southeast Asian and Central Asian regions. There are some limitations to our evaluations that should be noted. First, impact was not assessed. This would require a longer period of evaluation, which is beyond the scope of the current evaluation. Nevertheless, first, the current findings on applicability suggest potential impacts, which are to be investigated and confirmed by a follow-up evaluation study. Second, our findings are based on the limited number of GHRP courses implemented. This is mostly attributable to the limitations of the available time and resources for more courses, and thus it is expected that the early dissemination of the current evaluation could promote opportunities for further funding to increase the number of courses and provide an opportunity for evaluation on a larger scale. Third, given that only a limited number of participants are experiencing the course at this pilot stage, the findings are mainly qualitative and thus largely context-specific. There will be opportunities to complement with a more quantitative assessment when the course is rolled out across the regions by RTCs.

Nevertheless, there is momentum for the current GHRP course to further develop into international standards for public health research, with the support of a committed group of experts and a range of global health institutions, as well as evident demand among researchers in LMICs. Although an immense amount of evidence on health research standards and practices is available in the scientific world today, researchers in LMICs may often have no or limited access to universally recognized ethical standards and best practices in research, as well as latest developments in science. Hence, in these regions, a well-structured course that condenses the key principles, international standards, and best practices of public health research, highlighting ethics and quality, can contribute to meeting the existing demand for the development and strengthening of research and publication capacities.

However, to enable eventual development of the GHRP course into international standards to ensure the ethics and quality of health research, there is certainly a need to develop a consensus with a broader range of key stakeholders on the final set of GHRP standards and means for implementation. In parallel, a number of action points still require to be followed up to further strengthen the GHRP training program. First, there is a need to develop a facilitator guide to ensure further standardization of the course materials and teaching technique. Second, a sufficient number of trained facilitators need to be prepared. This will entail developing criteria for trainers, preparing a training of trainers session, and identifying the master trainers. Third, there is a need to design workshops to facilitate integrating elements of the GHRP course into existing post-graduate curricula and accreditation schemes. This effort will foster the institutionalization and ownership of the GHRP course in countries with existing capacity gaps. Beyond these steps for scaling up the GHRP training, there is certainly a need to develop a consensus with a broader range of key stakeholders on the final set of GHRP standards and means for their implementation.

Moreover, the course in Almaty demonstrated that, in certain regions there is still a high level of inequity in terms of access to and the uptake of health evidence and best practices as a result of language barriers. With a considerable amount of health information predominantly disseminated in English, countries with limited English fluency cannot benefit from the existing body of health evidence and practice. The experience of delivering a course in English with simultaneous translation into Russian, alongside translated training materials, raised the numerous issues that must be addressed if the course is given in other languages. First, in the short term, a high-quality translation of written material and involving professional simultaneous interpreters with proven record of working with public health material must be ensured. Second, in the long-term, local facilitators must be trained to deliver the course in regional languages (such as Russian, Spanish, and French).

The current GHRP course model that revolves around the principles of ethics and quality is evidently relevant, applicable, and well received by researchers in LMICs working on non-clinical trials health research. There is an urgent need to harness the momentum to promote high-quality and ethical health research in LMICs through scaling up GHRP training and further development of GHRP principles into international standards.

## Paper context

GCP guidelines have been the source of improvement in the quality of clinical trials; however, there are limitations to the application of GCP in the conduct of health research beyond clinical trials. GHRP training program has been developed to promote good practices in all health research involving human. This paper report results from piloting of GHRP training programs and highlights steps needed to scale up GHRP training and development of GHRP principles into international standards.
